# Enteric-type adenocarcinoma of the lung harbouring a novel *KRAS* Q22K mutation with concomitant *KRAS* polysomy: a case report

**DOI:** 10.3332/ecancer.2015.559

**Published:** 2015-07-28

**Authors:** Giulio Metro, Emanuele Valtorta, Annamaria Siggillino, Calogero Lauricella, Matteo Cenci, Vienna Ludovini, Elisa Minenza, Enrico Prosperi, Biagio Ricciuti, Alberto Rebonato, Alessandra Bassetti, Lucio Crinò

**Affiliations:** 1 Medical Oncology, Santa Maria della Misericordia Hospital, Azienda Ospedaliera di Perugia, Perugia 06156, Italy; 2 Niguarda Cancer Center, Division of Pathology, Ospedale Niguarda Ca’ Granda, Milano 20162, Italy; 3 Medical Oncology, Santa Maria Hospital, Azienda Ospedaliera di Terni, Terni 05100, Italy; 4 Department of Experimental Medicine, Pathological Anatomy and Histology Unit, School of Medicine, University of Perugia, Perugia 06156, Italy; 5 Department of Diagnostic Imaging, Santa Maria della Misericordia Hospital, University of Perugia 06156, Italy; 6 Medical Oncology, Narni Hospital, Narni (TR) 05035, Italy

**Keywords:** DNA sequencing, enteric-type adenocarcinoma, gene mutation, KRAS, non-small cell lung cancer

## Abstract

This case describes a novel *KRAS* Q22K mutation with simultaneous *KRAS* polysomy in a patient with advanced, enteric-type, adenocarcinoma of the lung. Despite the administration of systemic chemotherapy, the disease underwent rapid progression and led to the patient’s death in a short period of time. Such an aggressive clinical course suggests that, in this specific case, *KRAS* dependency was the major genetic driver of poor prognosis. Direct deoxy ribonucleic acid (DNA) sequencing of the *KRAS* gene allows for the detection of novel *KRAS* mutations, and it might be advocated in patients with advanced non-small cell lung cancer in view of the emerging role of *KRAS* as a potential therapeutic target.

## Introduction

Mammalian cells express three closely related, small proteins of 189 amino acids with a molecular weight of 21 kDa (p21), termed *KRAS*, *NRAS*, and *HRAS* [1]. Localised in the inner plasma membrane, they function as GTPases that promote different types of ligand-mediated signal transduction pathways involved in cell proliferation, differentiation, and apoptosis [2]. RAS proteins cycle between two conformational states. One is when they are in the guanosine triphosphate (GTP)-bound active state, which is promoted by the guanine nucleotideexchange factors (GEFs); another one is when they bound to guanosine diphosphate (GDP), the inactive form, which is induced by the GTPase-activating proteins (GAPs).

Importantly, human cancers are preferentially associated with mutations in the *KRAS* gene, which almost invariably result into a constitutively active, GTP-bound RAS protein with pro-oncogenic effects [3]. In lung adenocarcinoma, somatic mutations of the *KRAS* gene are relatively frequent, occurring in approximately 11% and 26% of cases from Asian and Western patients, respectively [4]. In the majority of cases, these mutations are missense mutations that introduce an amino acid substitution at codon 12, 13, or, less frequently, 61 [3]. Here, we present the case of a patient with an enteric-type adenocarcinoma of the lung harboring a novel mutation at codon 22 of *KRAS* (Q22K) with concomitant *KRAS* polysomy.

## Case report

**Figure 1.**
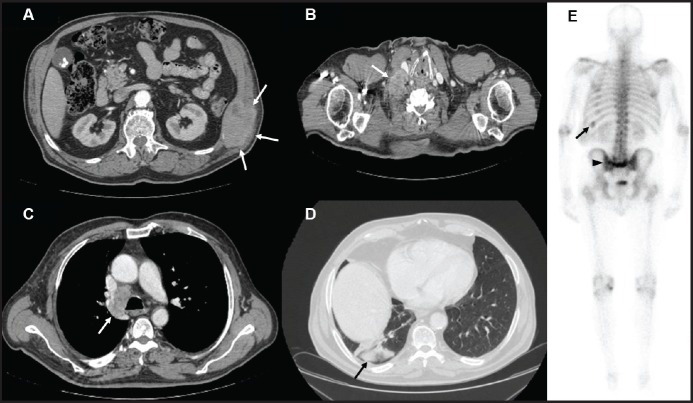
Contrast enhanced CT scan of case presentation. (A) osteolytic mass of the eleventh left rib (white arrows) invading surrounding soft tissues; (B) bulky lymph-nodal metastasis (white arrow) of the right posterior cervical space dislocating the right common carotid artery; (C) lower paratracheal, 4R according to IASLC, lymph-nodal metastasis (white arrow); (D) subpleural mass in the posterior segment of inferior right lobe; (E) Bone scintigraphy showing active pathological uptake of the left eleventh rib (black arrow) and of S2 vertebral body (black arrow head).

**Figure 2.**
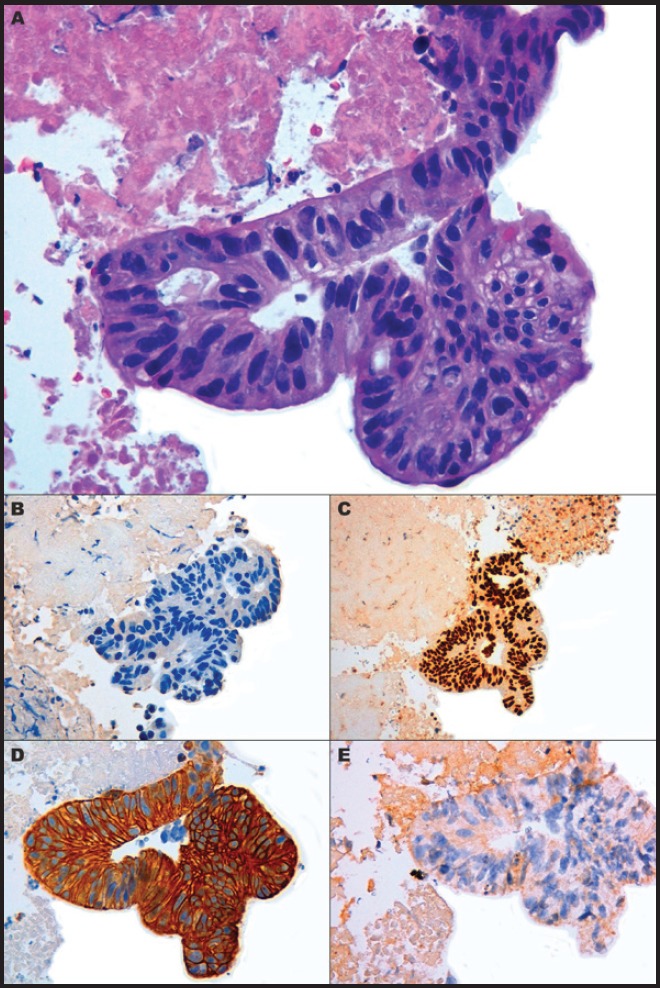
Microscopic presentation of the tumor showing enteric-type adenocarcinoma. (A) Cluster of neoplastic cells arranged in acinar pattern show atipical nuclei and clear cytoplasmatic droplets of mucin; (B) Immunohistochemically cells are negative to TTF-1; (C) Expression of CDX2 and (D) cytokeratin 7; (E) No immunoreactivity is evident for Cytokeratin 20. (A) Hematoxylin and Eosin, original magnification X40 - (B) X40 - (C) X20 – (D) X40 – (E) X40.

A 74-year-old male with a 25 pack/year history of smoking was referred to the Medical Oncology of the Narni Hospital (TR, Italy) because of a voluminous right laterocervical lymphadenopathy. Two months earlier, he had started complaining of severe pain in the sacral region. An incisional biopsy of the enlarged lymph-nodal mass was positive for the presence of metastasis from poorly differentiated adenocarcinoma with signet ring cell features. A full-body computed tomography (CT) scan confirmed a 60 mm large laterocervical lymphadenopathy, also revealing a right lung lesion, a rib metastasis, and a voluminous neoplastic involvement of the first sacral vertebra (Figure 1), whose extension went beyond the spinal canal, infiltrating the radicular fibres. In addition, the patient underwent an endoscopic study of the gastroenteric tract with oesophagogastroduodenoscopy and colonoscopy, both tests excluding the presence of a primary gastrointestinal cancer. Histologic revision from a pathologist (EP) of the Perugia Hospital (PG, Italy) supported the diagnosis of pulmonary enteric-type adenocarcinoma (Figure 2).

**Figure 3.**
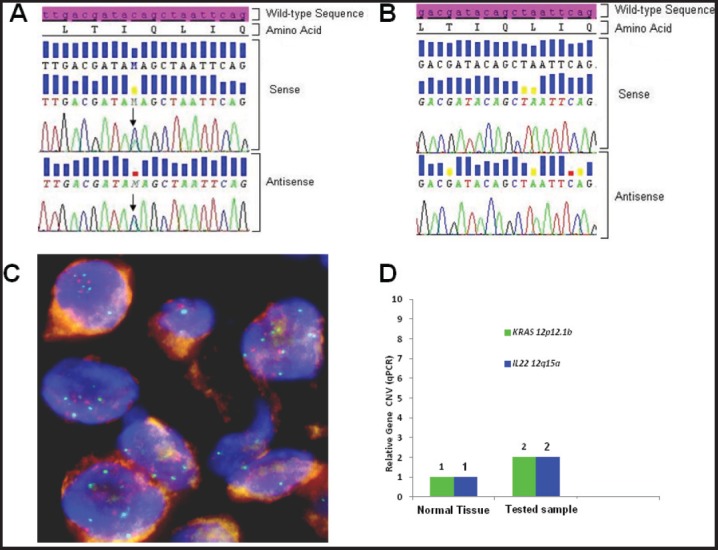
Biologic profile of the tumor. (A) Sequence analysis of the *KRAS* exon 2 performed on DNA extracted from microdissected area of paraffin-embedded tumor sections, shows a heterozygous point mutation: a C to A transversion (black arrow), resulting in a substitution of Glutamine, wild type (CAG) for Lysine (AAG) at codon 22 (p.Q22K, c.64C>A ). The mutation was named according to recommendations of the Nomenclature System for Human Gene Mutations. NCBI Reference Sequence: NM_004985.4 of *KRAS* gene was used as a reference; (B) Sequence analysis of the *KRAS* exon 2, performed on DNA extracted from peripheral white blood cells, shows the sequence wild type (CAG) resulting in normal glutamine at codon 22; (C) Dual colour FISH analysis (original magnification 60X) was performed using a CEP12 alpha satellite probe (12p11-q11) labelled in SpectrumOrange (Vysis, Downers Grove, IL. USA) and a BAC (Bacterial Artificial Chromosome) genomic probe RP11-707G18 (12p12.1) spanning an approximately 176 kb region encompassing the *K-Ras* gene, labelled in SpectrumGreen; (D) Representative data from *KRAS* and *IL22* relative quantification (RQ) using TaqMan Copy Number Assays run on ViiA7 instrument. The number of copies of the *KRAS* and *IL22* is determined by relative quantitation (RQ) using the comparative CT (ΔΔCT) method. This method measures the CT difference (ΔCT) between target and reference gene (RNase P), then compares the ΔCT values of test samples to a calibrator sample(s) known to have two copies of the target sequence. The copy number of the target is calculated to be two times the relative quantification.

Using somatic DNA extracted from the lymph-nodal biopsy, the sequence analysis for both epidermal growth factor receptor (*EGFR*) (exon 18 to 21) and *KRAS* (exon 2 and 3) genes was performed by nested polymerase chain reaction (PCR) and direct sequencing [5]. While no *EGFR* mutation was detected, a Q22K point mutation was described in the *KRAS* gene (Figure 3A). Such a mutation was not found in the peripheral white blood cells, thus indicating its somatic origin in the tumor (Figure 3B). Paraffin-embedded samples were then subjected to immunohistochemical (IHC) analysis for ALK (clone D5F3, cell signalling) and ROS1 (clone D4D6, cell signalling), which tested negative for both markers (not shown). To assess whether the *KRAS* Q22K mutation was accompanied by an increased *KRAS* gene copy number, *KRAS* FISH analysis was performed, which documented the presence of polysomy (≥ 4 copies in 10–40% of cells) for the *KRAS* gene (Figure 3C) [6]. In addition, quantitative polymerase chain reaction (qPCR) was carried out, which confirmed a copy number gain of 4 (Figure 3D) [7].

After receiving a single radiation dose of 8 Gy to the L4-S2 spinal tract, a decision was made to administer systemic chemotherapy with single-agent gemcitabine 1,000 mg/m^2^ days 1–8 every 21. A CT scan performed after three cycles of chemotherapy documented disease progression in the bone. On this basis, the patient was switched to second-line pemetrexed 500 mg/m^2^ every 21 days. However, after only two cycles of this regimen, the patient’s clinical conditions deteriorated rapidly, and he died because of progressive disease shortly thereafter.

## Discussion

To our knowledge, this is the first report of a *KRAS* Q22K mutation in lung adenocarcinoma. This very rare mutation has been described only in a few other cancers, namely large/small intestine, haematopoietic and lymphoid, central nervous system (CNS), and pancreas, with a reported incidence that has been consistently lower than 1% for each of the aforementioned malignancies [8]. In the present case, a *KRAS* Q22K mutation was detected in an unusual variant of pulmonary adenocarcinoma, such as the enteric type. Importantly, the clinical presentation of the tumour was crucial in order to ascertain the primitive lung nature of the cancer (Figure 1).

*KRAS* Q22K mutation consists of a C to A transversion substituting lysine (AAG) for normal glutamine (CAG) at the 22^nd^ amino acid residue of RASp21, which is structurally located between the GTPase domain and the effector domain binding GAPs [9]. As a result, the affinity of RASp21 for guanine nucleotide is influenced, which allows *KRAS* Q22K mutant to be stabilised into a constitutively activated GTP-bound form. Accordingly, preclinical studies have shown that cell lines expressing the *KRAS* Q22K mutation possess high *in vivo* oncogenic potential, higher than that of wild-type *KRAS* [9]. This finding is in line with the evidence that non-small cell lung cancer (NSCLC) cell lines transfected with *KRAS* Q22K mutants show elevated levels of RAS-GTP and downstream phosphorylated ERK, compared with wild-type *KRAS* [10].

Inconclusive data have been reported in the literature about the role of *KRAS* mutation as ‘driver’ genetic alteration in advanced NSCLC [11, 12]. Current knowledge only suggests that the presence of *KRAS* mutation is a negative predictor for treatment with an EGFR-TKI [13]. However, the heterogeneity of *KRAS* mutations in NSCLC limits the further applicability of this finding [14]. Nevertheless, in this specific case, the ‘driver’ role of *KRAS* Q22K mutation can be deduced from a few reasons. First, the mutation was confirmed to be somatic (Figure 1B). Secondly, some other ‘driver’ genetic alterations were excluded (i.e., EGFR, ALK, and ROS1), thus suggesting the presence of *KRAS* mutation as a single-mutated oncogene. Also, *KRAS* FISH and qPCR analyses showed polysomy of the *KRAS* gene, and preclinical models of *KRAS* Q22Kmutant tumors have shown even enhanced tumorigenicity in the presence of increased *KRAS* gene copy number (either *KRAS* amplification or *KRAS* polysomy) [9]. Accordingly, a worse prognosis has been suggested for patients who harbour a *KRAS* mutation simultaneously with an increased *KRAS* gene copy number > 3 [15]. Finally, as shown by the sequencing electropherogram (Figure 1A), *KRAS* mutant allele peak was equal to that of wild-type *KRAS*, which is in line with a previous report suggesting worse prognosis in the presence of *KRAS*-mutant allele greater than or equal to wild-type allele [16]. Taken together, these data strongly suggest that, in this specific case, the proliferation and survival of the tumour was largely sustained by *KRAS* Q22K mutation and simultaneous *KRAS* polisomy.

## Conclusions

The present case unveils a novel *KRAS* Q22K mutation in lung adenocarcinoma and suggests the potential driver role of this specific *KRAS*-mutant genotype when associated with increased *KRAS* gene copy number. Finally, it supports direct DNA sequencing as the ‘gold standard’ for the detection of *KRAS* mutations in NSCLC, since it allows the uncovering of uncommon mutations as opposed to ‘target’ methods (e.g., Pyrosequencing, TheraScreen), which reveal only predetermined *KRAS* gene mutations. This is of particular relevance in view of the ongoing clinical trials that are testing the effectiveness of molecules aimed at inhibiting the RAS/RAF/MEK/ERK pathways [17, 18], which could be offered as an alternative treatment approach to patients biologically selected for *KRAS* mutation.

## Conflicts of interest

The authors have no conflict of interest to declare.

## References

[1] Platz A (2008). Human cutaneous melanoma; a review of NRAS and BRAF mutation frequencies in relation to histogenetic subclass and body site. Mol Oncol.

[2] Stephen AG (2012). Dragging ras back in the ring. Cancer Cell.

[3] Prior IA, Lewis PD, Mattos C (2012). A comprehensive survey of Ras mutations in cancer. Cancer Res.

[4] Dearden S (2013). Mutation incidence and coincidence in nonsmall-cell lung cancer: meta-analyses by ethnicity and histology (mutMap). Ann Oncol.

[5] Ludovini V (2012). Optimization of patient selection for EGFR-TKIs in advanced non-small cell lung cancer by combined analysis of KRAS, PIK3CA, MET, and non-sensitizing EGFR mutations. Cancer Chemother Pharmacol.

[6] Smith G (2010). Activating K-Ras mutations outwith ‘hotspot’ codons in sporadic colorectal tumours—implications for personalised cancer medicine. Br J Cancer.

[7] Bardelli A (2013). Amplification of the MET receptor drives resistance to anti-EGFR therapies in colorectal cancer. Cancer Discov.

[8] COSMIC—Catalogue of somatic mutations in cancer http://cancer.sanger.ac.uk/cancergenome/projects/cosmic/.

[9] Tsukuda K (2000). A novel activating mutation of the K-ras gene in human primary colon adenocarcinoma. Biochem Biophys Res Commun.

[10] Janakiraman M (2010). Genomic and biological characterization of exon 4 KRAS mutations in human cancer. Cancer Res.

[11] Meng D (2013). Prognostic value of K-RAS mutations in patients with non-small cell lung cancer: a systematic review with meta-analysis. Lung Cancer.

[12] Metro G (2014). Clinical outcome with platinum-based chemotherapy in patients with advanced nonsquamous EGFR wild-type non-small-cell lung cancer segregated according to KRAS mutation status. Clin Lung Cancer.

[13] Mao C (2010). KRAS mutations and resistance to EGFR-TKIs treatment in patients with non-small cell lung cancer: a meta-analysis of 22 studies. Lung Cancer.

[14] Metro G (2012). Impact of specific mutant KRAS on clinical outcome of EGFR-TKI-treated advanced non-small cell lung cancer patients with an EGFR wild type genotype. Lung Cancer.

[15] Sasaki H (2011). Evaluation of Kras gene mutation and copy number gain in non-small cell lung cancer. J Thorac Oncol.

[16] Chiosea SI (2012). KRAS mutant allele-specific imbalance in lung adenocarcinoma. Mod Pathol.

[17] Jänne PA (2013). Selumetinib plus docetaxel for KRAS-mutant advanced non-small-cell lung cancer: a randomised, multicentre, placebo-controlled, phase 2 study. Lancet Oncol.

[18] Blumenschein GR (2013). MEK114653: a randomized, multicenter, phase II study to assess efficacy and safety of trametinib (T) compared with docetaxel (D) in KRAS-mutant advanced non–small cell lung cancer (NSCLC). J Clin Oncol.

